# The Structure Lacuna

**DOI:** 10.3390/ijms13079081

**Published:** 2012-07-20

**Authors:** Jan C.A. Boeyens, Demetrius C. Levendis

**Affiliations:** 1 Unit for Advanced Scholarship, University of Pretoria, Lynnwood Road, Pretoria 0002, South Africa; 2 Molecular Sciences Institute, School of Chemistry, University of the Witwatersrand, Jan Smuts Avenue, Johannesburg 0001, South Africa; E-Mail: demetrius.levendis@wits.ac.za

**Keywords:** golden ratio, molecular symmetry, spin function

## Abstract

Molecular symmetry is intimately connected with the classical concept of three-dimensional molecular structure. In a non-classical theory of wave-like interaction in four-dimensional space-time, both of these concepts and traditional quantum mechanics lose their operational meaning, unless suitably modified. A required reformulation should emphasize the importance of four-dimensional effects like spin and the symmetry effects of space-time curvature that could lead to a fundamentally different understanding of molecular symmetry and structure in terms of elementary number theory. Isolated single molecules have no characteristic shape and macro-biomolecules only develop robust three-dimensional structure in hydrophobic response to aqueous cellular media.

## 1. Introduction

Most quantum-theoretical concepts of chemistry have their origin in spectroscopy. The atomic spectroscopy of Kirchhoff, Bunsen, Fraunhofer and others resulted in the formulation of Balmer’s mysterious formula, the direct stimulus for the development of quantum mechanics. From molecular spectroscopy, which also dates from the 19th century, developed the concept of molecular symmetry, based on point-group theory. The concept of molecular structure was conjectured to bridge the gap between the two branches of spectroscopy. It is noted in passing that the discipline of quantum molecular theory is based entirely on this postulated, but unproven, concept of molecular structure.

The analysis of molecular crystals by X-ray diffraction is routinely accepted as final justification of the molecular-structure hypothesis, on assuming that individual molecules maintain their integrity and structure in the solid state. This assumption is demonstrably unjustified. The molecular unit projected from crystallographic analysis is best described as a rigid three-dimensional framework that connects point atoms and is stabilized by crystal-packing interactions. In the absence of such constraints the crystallographic point group no longer represents the shape of the more flexible molecule. Related techniques such as gas-phase electron diffraction define no more than a radial distribution function. Spectroscopists are well aware of the complications associated with defining the symmetry group of non-rigid molecules, but the chemist naïvely accepts that their primitive concept of molecular structure is consistent with the most fundamental theories of physics. It is not. A recently published history of quantum chemistry [1] never mentions the concept “molecular structure”.

The classic example of a non-rigid molecule is ammonia, which appears as a bipyramidal arrangement of interconverting polyhedra that reflects by tunneling through the plane of the central N atom [2]. It is pointed out [2] that:

As far as the quantum-mechanical formalism is concerned, ammonia is by far no exception. *Similar situations can be constructed for all molecules*.

They do not fit into the molecular-structure theme of traditional chemistry and are considered as “strange” states from a chemical point of view. *Neither chemical structures nor chemical bonds do exist any more*.

On the other extreme, molecules such as the adamantane-derived bispidine ligands have been demonstrated in synthetic studies to operate as rigid units with apparently well-defined classical structure [3]. We contend that the difference between ammonia and bispidine is one of degree only. In both cases the equilibrium molecular geometry is essentially non-classical. Only in the case of bispidine is there a striking correspondence with the structure predicted by classical concepts of valency.

Molecular symmetry is a classical concept based on the hypothetical model of point atoms held together by rigid chemical bonds. There is no experimental evidence for the existence of either point atoms nor chemical bonds. Both of these concepts are postulates to support the assumption of molecules with three-dimensional geometrical structure and molecular symmetry is the mathematical description of this structure. Alternatively molecular symmetry could be defined in line with more recent views on atomic and molecular structure.

A more appropriate theory, starting from the nature of electrons and atomic nuclei as wave-like distortions of four-dimensional space-time, characterizes a molecule as a 4D standing-wave structure in which individual particles do not have independent existence. The only meaningful symmetry of such a construct must be four- and not three-dimensional. It will be argued that classical molecular structure is the three-dimensional projection of non-classical four-dimensional molecular geometry.

## 2. Electron Theory

The point atom with mass *m_n_* is a remnant of the classically assumed ultimate philosophical limit to the subdivision of matter. It has been used in this form by Newton, Dalton, Mendeleév and others, until the end of the 19th century. Following Nagaoka, Rutherford and Bohr the concept of a structureless atom was abandoned and replaced by the planetary model of point-like electrons, with mass *m_e_* in orbit around a point-like heavy nucleus. With the advent of quantum mechanics the Bohr–Sommerfeld orbits gave way to chemical shells of probabilistic charge density—now defining the electron as a point particle! To account for the behaviour of electrons in diffraction experiments the kabbalistic notion of wave-particle duality was concocted, notwithstanding the wave-mechanical alternative proposed by Schrödinger.

As the particle model found its way into textbooks the electronic wave structure was completely forgotten, although the quantum-mechanical model never made any logical sense. In order to rationalize the probability function as representing a single electron it is necessary to integrate over all space. The electron associated with a given hydrogen atom, although likely to occupy the ground-state, has a finite probability to be on the moon. The chemists of the world have been content to live with this assumption for a hundred years. They have been brainwashed so thoroughly as not to tolerate any alternative suggestion that makes logical sense.

The most bothersome aspect of electronic quantum mechanics is the failure to account for the most prominent attribute of an electron, known as its spin. Efforts to artificially add a spin function to the three-dimensional state function of an electron have only been partially successful. A common remedy is often proposed through the assertion that spin is a relativistic phenomenon, but a conclusive proof has never been formulated. What has been demonstrated is that the spin function, known as a spinor, occurs naturally as an inherent feature of four-dimensional motion. In order to define an electron with spin it is therefore necessary to describe it as an entity in four-dimensional space-time. This would automatically rule out its definition as a point particle, noting that a point in four-dimensional space-time has, not only zero extension but also zero duration. It cannot exist.

With this realization the interminable debate about particle or wave becomes redundant and the remaining option is to consider the electron as a four-dimensional wave structure. In fact, Schrödinger’s equation is a modification of the general wave Equation. It is fashionable to state [4] that

… analogies between geometrical optics and classical mechanics on the one hand, and wave optics and quantum mechanics on the other hand … can only make the Schrödinger equation seem *plausible*; they cannot be used to *derive* or *prove* this equation.

Statements like this are standard textbook fare that derives from the Copenhagen campaign to discredit Schrödinger’s wave interpretation [5]):

(1) $$\begin{array}{c} \frac{\partial^{2}\Phi }{\partial x^{2}}+\frac{\partial^{2}\Phi }{\partial y^{2}}+\frac{\partial^{2}\Phi }{\partial z^{2}}-\frac{1}{c^{2}}\frac{\partial^{2}\Phi }{\partial t^{2}}=0\\ \left(\nabla^{2}\Phi =\frac{1}{c^{2}}\frac{\partial^{2}\Phi }{\partial t^{2}}\right) \end{array}$$

By defining *x*_0_ = *ict*, *etc*., this equation rearranges into

$$\frac{\partial^{2}\Phi }{\partial x_{1}^{2}}+\frac{\partial^{2}\Phi }{\partial x_{2}^{2}}+\frac{\partial^{2}\Phi }{\partial x_{3}^{2}}+\frac{\partial^{2}\Phi }{\partial x_{0}^{2}}=0$$

*i.e.*,

(2) $$\left(\partial^{2}\Phi =0\right)$$

The solution of Equation 2 has the hypercomplex form

Φ=a+ib+jc+kd

known as a quaternion or the spin function. The coefficients *i*, *j*, *k* are generalizations of $\sqrt{-1}$ with the rule of composition: *i*^2^ + *j*^2^ + *k*^2^ = *ijk* = −1.

In order to obtain Schrödinger’s solution it is necessary to separate the space and time variables on solving Equation 1. The result of this is to describe the electron as a three-dimensional object without spin. The consequence is that the three-dimensional angular momentum defined by Schrödinger’s equation is not a conserved quantity and hence inadequate as a basis on which to construct molecular symmetry functions. The conserved quantity is traditionally defined as ***J*** = ***L*** + ***S***, a solution of Equation 2.

In the quaternion representation an electron can no longer be considered as an entity that moves through space, but rather as a distortion of space-time, or the vacuum. A vivid visualization was proposed by Weingard [6]

… conceiving of the motion of particles on analogy with the motion of the letters on the illuminated news sign in Times Square. The letters move, not by anything moving, but by the sequential blinking of the lights of which the sign is composed. Similarly, we can conceive of the motion of a particle as the motion of a geometrical property. Regions of space do not move, but the geometry of adjacent regions change in such a way that a pattern of geometrical properties—the pattern we identify with a certain particle—moves.

In quaternion terminology such a geometrical pattern may be described as a standing four-dimensional spin wave. Such a wave packet is flexible, by definition, and adapts to the geometry of its local environment. The size of a wave packet in space-time is restricted by the limited time component that prevents indefinite extension. The hydrogen electron can no longer be on the moon. The topology of the wave packet manifests, in addition to spin, also as characteristic charge and mass.

On interaction with a proton, a spin wave of opposite charge and high mass, the electron envelops the proton in a hyperspherical shroud. Assuming that waves of similar topology readily coalesce into larger composite waves, the formation of heavier atoms appears as a natural process. All interactions and rearrangements consist of finding an equilibrium among merging patterns. The only feature to explain is the origin of the elementary patterns such as electron, proton and neutrino.

The answer is provided by the theory of general relativity. We note in passing that the Lorentz transformation that defines special relativity amounts to a complex rotation in four-dimensional space-time, with the same structure as the quaternion spin function [7]. Wave mechanics and relativity collapse into a single theory [8]. The additional consideration that creates general relativity is formulation of the theory in non-Euclidean space-time. The resulting field equations are of the form

$$G_{\mu \nu }=R_{\mu \nu }-g_{\mu \nu }R=kT_{\mu \nu }$$

In words, the curvature tensor *G_μν_* balances the energy-stress tensor *T_μν_*, which may be interpreted as distortions (matter) in space-time. This means that Euclidean (flat) space-time has *T_μν_* = 0 and hence contains no energy or matter. As space-time curves, elementary wrinkles develop and coalesce into atomic matter and eventually into macroscopic massive objects that cause increased local curvature of space-time, recognized as a gravitational field.

## 3. Atomic Structure

The empirical Periodic Table of the Elements is widely accepted as the major contribution of chemistry to science, and correctly so. Whereas the Bohr–Sommerfeld atomic model could be demonstrated to be consistent with the periodic model by invoking the empirical exclusion principle of Pauli, the Schrödinger wave-mechanical model can only partially account for elemental periodicity. Two reasons for the discrepancy are the neglect of spin and the assumption of zero interaction with the environment as in Euclidean space-time.

A few years ago it was demonstrated [9] that, on specifying the local curvature of space-time by the ubiquitous golden parameter, $\tau =\frac{1}{2}(\sqrt{5}-1)$, details not only of elemental periodicity but also of the general periodicity of all stable nuclides may be mapped by elementary number theory as a function of space-time curvature. It is of special interest to note that this periodic function extends in a natural way to include antimatter in an involuted arrangement that strongly supports a projective topology of space-time.

Of more immediate relevance is that appearance of the golden mean indicates a self-similar relationship between atomic structure, botanical growth, patterns in the solar system and spiral galaxies. In response it could be shown [10,11] that extranuclear electron density in all atoms is simulated by golden logarithmic-spiral optimization. The result is a spherical distribution of charge in the form of a standing wave around the nucleus. The distribution mirrors the best quantum-mechanical models in the form of the arrangement predicted by Thomas–Fermi statistics [12] as well as self-consistent-field Hartree–Fock calculations [13] of atomic structure.

## 4. Chemical Interaction

**Figure 1:**
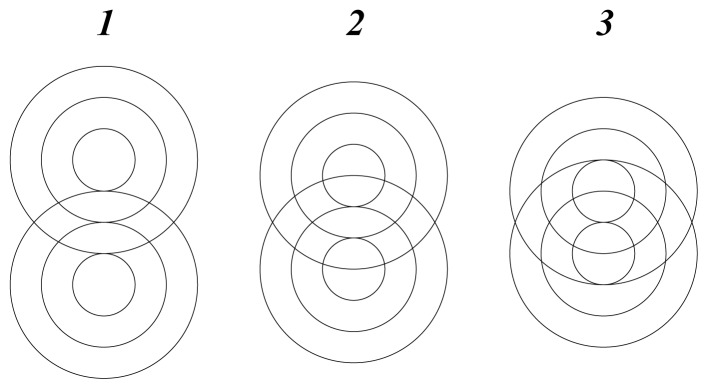
Interactions of different order.

The classical straight-line representation of a chemical bond poorly describes the mode of interaction between spherical wave structures. A more likely model is pictured in Figure 1 as the idealized interference pattern of two wave systems.

The consecutive frames may be interpreted as schematic representations of homonuclear covalent interactions of increasing order. To distinguish between different modes of overlap, frames 1 and 3 could represent integer and frame 2 half-integer order.

The relative nuclear positions implied by the two-dimensional drawing of Figure 1 are not necessarily fixed, as the atomic waves may rotate around each other in all directions, without disturbing the interference pattern. This conclusion is consistent with the standard assumption of spherical molecules in the study of gas kinetics [14].

**Figure 2:**
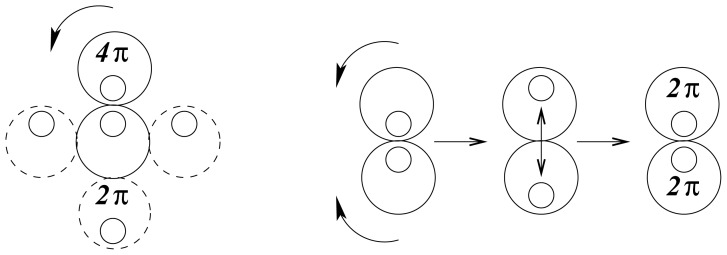
Spherical and geared rotation of spheres.

There are two possible modes of rotation, shown schematically in Figure 2. Either one sphere rotates, by rolling around the other, or the spheres rotate together like mating gears. The geared rotation does not change the relative disposition of the spheres and corresponds to the classical model of a vibrating diatomic molecule. The other mode of rotation creates a situation without a fixed axis of symmetry. It amounts to rotation around a point as described by the quaternion spin function. It requires a rotation of 4*π* to restore the initial configuration and represents an element of four-dimensional symmetry, which does not occur in three dimensions.

In this mode the cyclic disposition of atomic cores fluctuates with the spin function, consistent with the interaction between wave structures being subject to spin pairing. The hyperspherical molecular symmetry may be visualized as the interpenetration of two wave packets in spherical rotation such that the equilibrium separation of their centres of mass defines a vibrating interatomic distance. Generalization of this pattern suggests that atomic cores in multi-atomic molecules are confined, with restricted freedom, within a sea of valence electrons.

In the case of heteronuclear interaction the interference patterns must obviously be of lower symmetry, but in four dimensions it has the same hyperspherical symmetry as before. As more atoms participate in the formation of larger molecules a unique holistic interference pattern characterizes each equilibrium arrangement. The removal or displacement of any atom in the molecule from/by another affects the entire wave pattern, and hence the molecular symmetry, in a non-trivial way.

### 4.1. The Valence State

Atoms do not interact spontaneously to form molecules under all circumstances. Chemical theory assumes that interaction in a reacting system only commences under specific thermodynamic conditions that define a characteristic valence state for the system. This happens when a single activated electron becomes decoupled from its atomic core and free to interact with a similarly activated electron from another atom, as shown in Figure 1. The wave patterns of Figure 1 therefore only refer to a single pair of activated valence electrons and not the composite electronic wave of the core.

The decoupled valence electron behaves like an elementary charge, which is smeared out uniformly over a sphere of radius *r*_0_, known as the characteristic ionization radius (not to be confused with ionic radius) [15] of the atom. Electronic charge density as calculated by the spherical wave model of an atom [11] is distributed in the form of annular segments, separated by nodal surfaces. In order to specify the ionization sphere of an atom, the total charge in the outermost segment is normalized over a sphere of constant charge density, with a radius defined as *r*_0_. The valence electron in the activated stationary state with spherical symmetry has neither kinetic nor classical potential energy and its energy of

$$E_{g}=\frac{h^{2}}{8mr_{0}^{2}}$$

which represents quantum-potential energy (or chemical potential of the valence state) of

$$V_{q}=\frac{\hbar \nabla^{2}R}{2mR}$$

is simply related to the classical electronegativity [16], $\chi =\sqrt{E_{g}}$, with *E*_g_ in eV.

All covalence interaction parameters such as interatomic distance, bond order and dissociation energy should therefore be simple functions of *r*_0_. Steric distortion of the equilibrium molecular arrangement is resisted by the interference pattern that requires either integer or half-integer bond orders. Such resistance should correlate with spectroscopically measured harmonic stretching force constants. In principle all characteristics of covalent interaction could therefore be calculated directly from atomic ionization radii as the only parameter.

### 4.2. Covalence

**Figure 3:**
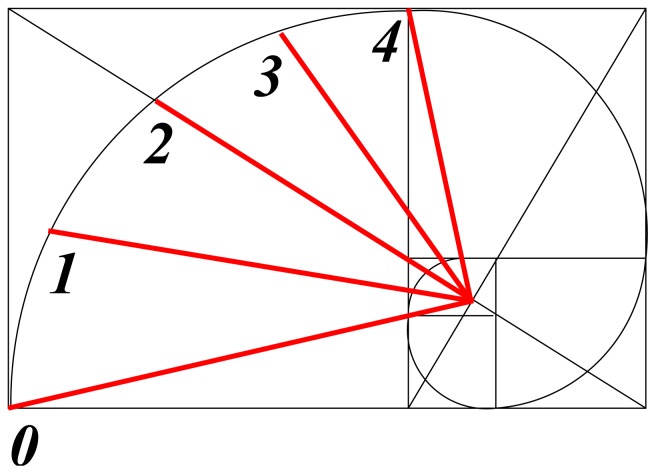
Golden-spiral optimization of bond order.

Although the definition of bond order as the extent of overlap between interfering waves is completely different from the classical concept, interatomic distances predicted by the wave model are remarkably similar to experimental estimates of empirically assumed bond orders. In terms of the wave model all interatomic distances, *d*, of homonuclear diatomic interactions of the same order are linear functions of ionization radius,

$$d=k_{b}r_{0}$$

where *k_b_* is characteristic for order *b*. In the same way that atomic electron distribution, as a standing wave, is predicted by logarithmic-spiral optimization, bond-order coefficients, *k_b_*, are specified in the same way, as shown in Figure 3, from [17]. It is instructive to note that *k_b_* varies from unity to *τ* for *b* = 0 to 4.

For heteronuclear interaction the relationship between interatomic distance and bond order is more complicated, but in all cases the golden ratio is involved. By the use of predicted bond orders and interatomic distance, dissociation energy and stretching force constants are next calculated directly from ionization radii and integral powers, *τ^n^*, of the golden ratio.

Other alternative approaches to the problem of covalence have appeared in the recent literature [18–22]. The main thrust of this research is to find an alternative to molecular-orbital theory in terms of variables such as chemical action and chemical hardness, based on their relationship with electronegativity and defined by various techniques, including semi-classical path integrals and second quantization, culminating in the definition of *bondons* to represent the electron pairs of covalent interaction. Final synthesis of these ideas into a working model of covalence seems to converge towards an understanding based on the role of the quantum potential and the golden ratio [23].

To cut a long story short we repeat that the characteristics of electrons, atoms and molecules are intimately related to the golden parameter and in order to understand molecular structure and symmetry we find it necessary to establish why this should be the case. At the most fundamental level we suspect the golden ratio to function as a critical characteristic of the general curvature of space-time. In order to explore this possibility it is necessary to consider a few relevant aspects of cosmology. We start from classical cosmology with a known link to the golden ratio, which Johannes Kepler, on cosmological grounds, referred to as the *divine proportion*.

## 5. Cosmic Self-Similarity

The cosmology of Pythagoras [24,25] (and of Kepler), based on natural numbers, can be summarized briefly as follows:

The cosmic **unit** is polarized into **two** antagonistic halves (male and female) which interact through a **third** irrational diagonal component that contains the sum of the first male and female numbers (3 + 2), divides the **four**-element (earth, water, fire, air) world in the divine proportion of $\tau =(\sqrt{5/4}-1/2)$ and sounds the music of the spheres.

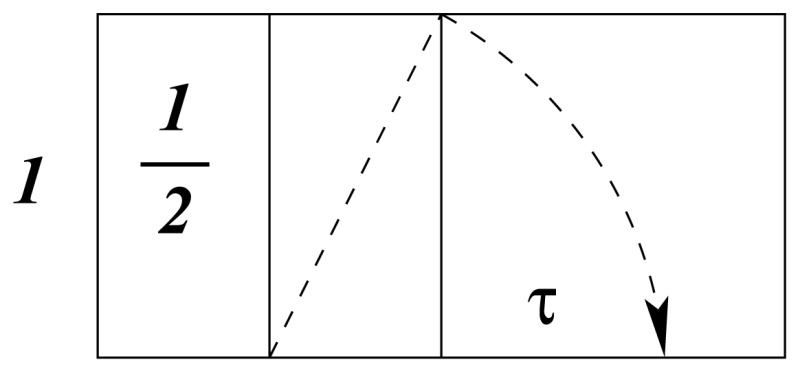

Translated into modern terms [26]:

The cosmic whole consists of a world and an anti-world, separated by an interface that creates all matter as it curves through four-dimensional space-time, with an involution, described by the golden ratio *τ*, which puts the universe into harmonious perspective of projective geometry.

For the benefit of prospective readers not familiar with projective geometry, a short introductory primer summarizes some details and Kepler’s contribution in the Appendix.

It seems that a relationship between cosmic structure and the golden ratio has been surmised for millenia without understanding its cause. Projective cosmology now suggests that the common factor is space-time curvature—a complete unknown. However, the prominent role of the golden spiral (Figure 3) could provide this insight. By construction, the golden spiral is made up of circular segments that fit diagonally into touching squares with side lengths in golden ratio. In each square a measure of the curvature follows from the ratio of arc length (*πr*/2) to the length of the diagonal chord ( $\sqrt{2}r$), *i.e*., $\pi /(2\sqrt{2})=1.111≃\sqrt{5}/2$. The agreement is not exact, but seductive (It is equally close to the ratio of 1.1115 said [27] to “keep popping up more frequently than coincidence would **seem** to allow”. The king’s chamber in the great pyramid is a 1:2 rectangle with its ceiling at the height of half its long floor diagonal, $\sqrt{5}/2$. [28]). It provides sufficient grounds to assume that cosmic self-similarity is a manifestation of general space-time curvature, resulting from cosmic projective geometry, of $\sqrt{5}/2$, with respect to Euclidean tangent space.

We emphasize the similitude between Pythagorean and projective cosmologies to highlight the importance of self-similarity for the understanding of molecular symmetry. Noting that [29]

… space itself has a structure that influences the shape of every existing thing,

molecules, like space-time itself, would be shaped in four dimensions. This is referred to as non-classical molecular shape as distinct from the traditionally assumed three-dimensional classical shape. The closest accord between classical and non-classical molecular structures occurs in close confinement as in a crystal lattice. The biggest difference is in free interstellar space where any stable molecule is of highly symmetrical shape. As such a molecule finds itself in more crowded environments, as in a molecular cloud, each interaction reduces its symmetry according to Goldstone’s theorem [30]. Likewise, reduced temperatures and increased pressure cause further symmetry breakdown until the molecule in the crystalline state at 0 K has the least symmetry.

It becomes problematic to decide which of these is the true molecular symmetry. The 0 K classical structure which is used invariably in spectroscopic analysis is probably the worst possible choice. The reason why it is a good practical model is because spectroscopic analysis relies mainly on internal parameters such as interatomic distance, described correctly by the classical structure. Molecular shape and symmetry are not predicted by molecular spectra.

Without this knowledge the vibrational symmetry of molecules becomes as meaningless as the feckless caricature of statically rigid three-dimensional molecules.

## 6. Molecular Symmetry

Nowhere in nature is there exact symmetry, only equilibrium. The reason is that the symmetry of any object depends on the symmetry of its environment, which is nowhere rigorously isotropic. It is safe to conclude that there are no symmetrical molecules except at 0 K, which is unreachable. As a mathematical concept however, symmetry is precisely defined by group theory and we hear that [31]

… the number and kinds of energy levels which an atom or molecule may have are rigorously and precisely determined by the symmetry of the molecule or of the environment of the atom.

This statement can only refer to a hypothetical molecule considered as a static array of point atoms. Knowing the number and kinds of energy levels of such a molecule is of no chemical interest. In fact, there is only the single ground-state energy level. In systems of chemical significance group theory has no function unless molecular symmetry is already known empirically. One may ask, as in the classic hamburger commercial: “ where is the beef?”

Without any knowledge of molecular shape or symmetry all that remains is the connectivity pattern which is directly commensurate with the 0 K classical structure and symmetry *via* the techniques of classical molecular mechanics. In principle it is possible to progress from here to situations of chemical importance by modelling the effects of thermodynamics on classical bond parameters. Considerable progress has been achieved by simulations of this type, known as *molecular dynamics*. However, commentators [32] who proclaim the power of quantum mechanics to predict the structure, symmetry and chemical properties of non-existent chemical compounds can safely be ignored.

Chemistry has a long way to go towards an understanding of the four-dimensional shape and symmetry of molecules. A useful first step would be to admit the inadequacy of three-dimensional quantum mechanics, which cannot deal with four-dimensional wave phenomena such as the electromagnetic field and the solutions of Equation 2. It is encouraging to note that pedagogical trends in this direction [33,34] are gaining momentum. These authors object to the way in which chemical bonding is understood and taught, although unaware of the root cause of their irritation. The irksome orbital concept and the very idea of a chemical bond are relics of extravagant efforts to develop quantum-mechanical arguments from classical molecular structures. The result is a mess. As remarked by Sheldon Goldstein [35]:

… it is not unusual when it comes to quantum philosophy, to find the very best physicists and mathematicians making sharp emphatic claims, almost of a mathematical character, that are trivially false and profoundly ignorant.

The performance of chemists is even worse—they accept the claims of the physicist at face value, then insert their classical model as a quantum concept, by the simple device of describing complex functions as real hybrid orbitals [10]. The implied violation of the exclusion principle is considered a small enough price to pay for the recognition of classical molecular structure and symmetry in quantum chemistry. It means that in reality there is no conceptual support for molecular symmetry, the way it features as a pillar of theoretical chemistry. The whole scheme is self-contradictory—in order to construct *sp* hybrids the orthogonality of *s* and *p* functions, which means zero overlap, is simply ignored.

Equally remarkable is the way in which molecular spectroscopists keep up the pretence of a quantum theory, despite the well-known fact that the assignment of group frequencies is independent of the assumed molecular structure and symmetry. Without classical structure there is precious little left of molecular quantum theory and symmetry. To fill this hiatus would require a complete reformulation of the problem.

It is, first of all, necessary to appreciate the four-dimensional nature of molecules. The symmetry of such a molecule is to be understood, by analogy with the three-dimensional VSEPR model, as resulting from the equilibrium arrangement of various wave structures that minimizes the spin function. Molecules with non-zero residual angular momentum are optically active. All others project into three dimensions with spherical symmetry. It should be clear that this 3D projection cannot be static as the time coordinate of space-time, as perceived in projection, would be non-zero. This means that the shape of a free achiral molecule represents the average of a spherically pulsating configuration.

The spherical symmetry could break down if the molecule, when set into axial rotation, as in a microwave field, exhibits a dipole moment. In an applied electric field Stark modulation permits spectroscopic measurement of the dipole moment. Infrared and Raman spectra arise from molecular vibrations induced by further breakdown of symmetry. Molecular spectra simply do not reveal the structure or symmetry that molecules do not possess. Any symmetry argument that enters into the interpretation of molecular spectra arises from structure due to interaction with electromagnetic fields in the environment, becoming more pronounced in condensed phases, including solutions.

Three-dimensional structures inferred from NMR spectra and X-ray crystallography are purely classical and confirms no more than chemical connectivity patterns. The frequent occurrence of molecular rearrangements shows that these patterns are not necessarily invariant. It is of interest to note that the complete molecular eigenstate, even of the Born–Oppenheimer Hamiltonian of complex molecules, signifies spherical symmetry [36]. Structured molecules are therefore undefined also in three-dimensional wave mechanics. What is popularly known as molecular structure and symmetry are purely classical concepts. This means that the fashionable proclivity to relate chemical property to molecular structure is a sterile pursuit. Apart from its structure, a classical molecule has no other properties and a free molecule, which exhibits the full range of chemical properties, has no structure.

### Molecular Shape

Before the ideas outlined here can be incorporated into a meaningful theory of chemistry it is necessary to contemplate the nature of a non-classical four-dimensional molecule. For most chemists this would imply a major paradigm shift; away from their traditional view of rigid three-dimensional molecular quantum objects. The enormity of the proposed paradigm shift is underlined by statements from leading theoreticians such as [37]:

There is no such thing as spacetime in the real world of physics … It is an approximation idea, an extremely good approximation under most circumstances, but always only an approximation.

The first obstacle to overcome is the counter-intuitive notion of space-time entanglement that implies a temporal component to space variables. Projection into three-dimensional space separates space and time coordinates to create the illusion of an object in isotropic three-dimensional motion. This means that a hyperspherical object appears as a pulsating spheroid in three-dimensional space, if all environmental effects are ignored. This model describes a molecule in intergalactic space. By analogy with Rydberg atoms [38] it should behave as a *Rydberg molecule*, in which the distance scale between interacting units is highly inflated. This implies an increase in atomic (and molecular) size, ionization radius and interatomic distance, with concomitantly decreased dissociation energy and molecular stability. Not surprisingly only the most stable diatomic molecules H_2_, CO, N_2_, *etc*., have been observed [39]. Macromolecules would simply fall apart in intergalactic space. In interstellar dark molecular clouds (e.g., the horsehead nebula) with high concentrations of molecular material and dust, more complex species like simple amino acids have been recorded.

To develop these ideas into useful computational models is not a straightforward exercise; for the time being it is difficult to see how it could be developed beyond the classical model of pairwise interaction. The only topic of immediate interest appears to be the spontaneous folding of macromolecules with biological function into characteristic single conformations. As revealed by crystallographic analyses the overall structure of proteins is remarkably compact, at about the same level as the crystals of small organic molecules [40], which means that a sensible relationship between their function and classical molecular structure could be established theoretically. However, the simulation of the reversible folding and unfolding of protein molecules has been conspicuously unsuccessful.

Probably the most remarkable feature of protein folding is the observation [41] that the unfolding transitions are well established to be generally two-state at equilibrium, with only the fully folded and random unfolded states populated substantially. Partially-folded intermediates are relatively unstable. As remarked by Thomas Creighton [40]:

… simple estimates of the number of conformations possible with even a small polypeptide chain of, say, only 50 amino acid residues imply that an astronomical length of time, many orders of magnitude longer than the age of the universe, would be required for all of them to be sampled randomly. Consequently, the folding process must be directed in some way …

In our biased opinion the curvature of space-time is one possible factor that could direct such specific action. It is noted that in many proteins the primary polypeptide chain pursues a moderately straight course across the entire breadth of the structure and then turns to the other side, without getting knotted or entangled. Domains of uniform secondary structure (*α*–helix, *β*–sheet, *etc*.) may be predicted from the primary sequence and embedded structural elements that cause reverse turns in the surface of the protein ensure the eventual globular structure. However, since all biomolecules operate in aqueous environments, the influence of space-time curvature on molecular shape is effectively masked by hydrophobic interaction, which means that all non-polar amino-acid side chains are buried in the interior of the globule, with polar groups exposed in the hydrophilic surface; in exact analogy with the formation of micelles in soap solutions. The crucial consideration towards elucidation of protein folding therefore is the distribution of amino acids with the alternation of polar and non-polar regions required for the formation of globular micelles. The key to this is encoded in the primary amino-acid sequence, as generated by the evolution of life. Similar conclusions, from the opposite point of view [42], are summarized by the statement:

Water is an integral part of biomolecular structural organization, and is central to the assembly and three-dimensional shape of proteins and nucleic acids.

## 7. Conclusions

We have to conclude that the shape and symmetry of free molecules cannot be related to space-time topology. Molecules, small enough to persist in free space, are too small to develop a characteristic shape and macromolecules, large enough to exhibit three-dimensional structure, adopt their characteristic shape in response to interaction in condensed phases. It is only in those cases where such molecules are incorporated as building blocks in biological growth structures that the familiar Fibonacci patterns, characteristic of space-time topology, become noticeable [10,29]. The question of a robust structure for medium-sized molecules remains an open one and constitutes a serious lacuna in the interpretation of atomic and molecular spectra.

## Figures and Tables

**Figure A1:**
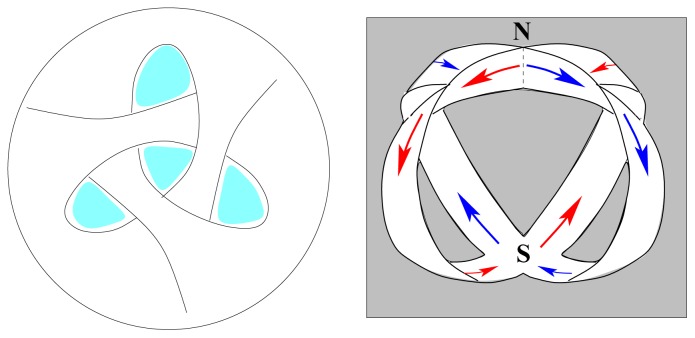
(**a**) Drawing of Boy’s surface [43]; (**b**) Intersecting Möbius bands.

## Appendix

### A. Projective Geometry

Any mathematical proof proceeds by way of deductive reasoning, which means that any fallacious conclusion or self-contradiction can be traced back to some fallacious axiomatic assumption. In Euclidean geometry one deals with one-dimensional straight lines and flat two-dimensional planes. A straight line is axiomatically created at the intersection of two flat planes, whereas the intersection of two straight lines defines a zero-dimensional point. By another axiom it is assumed that parallel straight lines or planes do not intersect. Certain straight lines or zero-dimensional points remain undefined by this reasoning, unless one of the axioms is abandoned. This dilemma was appreciated by Euclid, but no obvious resolution could be reached at the time.

With the advent of non-Euclidean geometries came the realization that, by giving up the axiom of parallel lines and planes a more general geometry, without exceptions could be formulated. The chosen remedy is to assume that two lines, which appear to be parallel, when extended indefinitely, intersect at infinity. As the two lines can be extended in opposite directions they must obviously intersect twice, at plus and minus infinity, which again contradicts the axiom that two straight lines intersect in a single point. The only way to avoid this conclusion is by assuming the two points of intersection to coincide and define a single point at infinity. This basic assumption of projective geometry can clearly not be satisfied in a Euclidean plane, even when extended indefinitely. The two points can only be brought into coincidence in a curved, so-called non-Euclidean plane. Since the argument is valid for parallel lines in all directions the implied curved surface, which defines projective topology, cannot be mapped in three dimensions without intersecting itself.

For better visualization of the situation consider the equivalent of straight lines between two points in the surface of a sphere, which is defined as a segment of the great circle that connects the points. Now there is another anomaly—two great circles intersect twice, at antipodal points. To avoid this problem the two points are identified as one to produce a geometrical construct with the same projective topology as before, known as a projective plane.

The projective plane is hard to visualize because it cannot be defined in three dimensions without intersecting itself. An artists impression of a model, known as Boy’s surface, as a visualization of the projective plane is shown schematically in Figure A1a. A more familiar visualization is provided by a Möbius strip, which is a segment sliced from the projective surface. Figure A1b shows two Möbius bands in the surface of a sphere, intersecting at the north pole. Adding more of the same, all intersecting in the same way, the entire spherical surface will eventually be covered on both sides by a single continuous surface that defines the projective plane. Like a single Möbius band the projective surface is created by a twist, known as an *involution*.

Although the projective plane cannot be embedded in three dimensions it is the most likely topology for a closed four-dimensional universe. Human beings are conditioned evolutionary to interpret the world in terms of three-dimensional Euclidean space, which may be considered as tangent to the underlying four-dimensional curved space-time. Those readers who are interested in the origins of projective geometry are referred to Stillwell’s [44] elementary discussion of Kepler’s seminal views on conic sections as projections of the circle. It is instructive to note that analysis of the group structure of the hypersphere  [45] defines, not only projective space, but also quaternion multiplication and spherical rotation, referred to as the “plate trick”.
